# Editorial: Current Issues in Perceptual Training: Facing the Requirement to Couple Perception, Cognition, and Action in Complex Motor Behavior

**DOI:** 10.3389/fpsyg.2019.02680

**Published:** 2019-12-03

**Authors:** André Klostermann, David L. Mann

**Affiliations:** ^1^University of Bern, Institute of Sport Science, Bern, Switzerland; ^2^Department of Human Movement Sciences, Amsterdam Movement Sciences and Institute of Brain and Behavior Amsterdam, Vrije Universiteit Amsterdam, Amsterdam, Netherlands

**Keywords:** perception-action coupling, sport science, performance, learning, transfer

In highly competitive sports, very little often separates the winner from their opponents. Sport scientists from around the world strive to uncover what it is that characterizes superior performance in these sports and it is these outcomes that hold the potential to further our understanding of human behavior and to optimize the training of skilled and developing athletes alike. Over recent decades, research has shown that *perceptual-cognitive skills* form an integral component of elite performance. More specifically, elite athletes are characterized by superior anticipatory and decision-making skills, are better able to recall sport-specific patterns, and show unique task-specific gaze behaviors (for an overview, see Mann et al., 2007). Studies have shown that perceptual-cognitive training can be effective to improve perceptual-skill and result in improvements in on-field performance (Farrow et al., 1998; Williams et al., 2002; Hopwood et al., 2011).

Despite the early promise shown for perceptual training, the wide variety of different training approaches and experimental designs adopted when evaluating training has resulted in a somewhat haphazard and unsystematic approach that makes comparisons between different approaches difficult (though see Abernethy et al., 2012). This includes inconsistencies in the training duration, frequency, and inclusion of tests of skill retention and transfer. Moreover, there is a lack of clarity about the degree to which perception and action should be coupled during training. Research has predominantly examined simplified training (and testing protocols) that fails to replicate the tight coupling between perception and action that would typically be present in the performance environment (i.e., designs lack *representativeness*). This is important because there is reason to question whether perceptual training would result in transfer if training does not incorporate the (motor) responses, the (visual) stimuli, and the perceptual function required when performing the real-world task (Hadlow et al., 2018).

Given the uncertainty about the training approaches most suitable to improve performance, this Research Topic sought to provide an overview of the past, present, but specifically the future approaches that may be suitable for perceptual training in sport. In doing so, the Research Topic was established to showcase current theoretical and experimental investigations. Scientists investigating all forms of perceptual training were approached and invited to take part, including those who advocate sport-specific approaches through to those that support more generic forms of vision and cognitive training. In the end, 13 papers from a variety of research groups around the world contributed to the Research Topic. Here we summarize those papers, categorizing them into those that tackle questions related to *perceptual-training interventions, task representativeness*, and *perception-action coupling*.

## Perceptual-Training Interventions

Gray in an ambitious longitudinal study tested the degree of learning possible in baseball batting following training in a virtual environment (VE). To this end, he compared four different groups who practiced either using adaptive VE batting-training (based on challenge-point theory), additional VE batting-practice, additional regular batting-practice, and regular-practice only. The adaptive VE batting-training group outperformed all other groups in the majority of outcome measures and, additionally, showed superior on-field batting performance in the season following the training. This represents one of the first studies demonstrating improvements in on-field performance following training in virtual reality.

Panchuk et al. instead trained athletes using immersive video footage but found only mixed results. They reported that immersive video training improved the decision-making of elite youth basketball players when later tested in the immersive environment, but that there was only limited transfer on-court.

North et al. took a more classic approach to compare the benefits of verbal-guidance and visual-guidance when training pattern recognition in soccer. Results showed that both training interventions improved pattern recall, but that the guidance provided no additional benefit beyond what was possible when simply viewing the same video sequences. Moreover, none of the groups improved their anticipatory ability following training, questioning the link between pattern recall and anticipatory skill.

Schorer et al. investigated the potential benefits of computerized pattern-recall training in combination with normal field-training in soccer. They found some evidence that, when tested with computerized test stimuli, in particular at retention, the experimental group outperformed the active control groups.

Two training studies investigated the potential benefit of blurring vision to enhance perceptual learning. Ryu et al. showed that participants who trained watching video footage containing low-spatial frequencies were less susceptible to deceptive actions when anticipating shuttle shot directions in badminton. Similarly, van Biemen et al. demonstrated a superior capability to distinguish dives from fouls after highly skilled football referees trained while watching blurred video footage of similar scenarios.

Finally, Harris et al. performed a systematic review to investigate the usefulness of commercial generalized cognitive training devices. In summary, they revealed good evidence only for the near transfer of these training devices, with limited evidence of far transfer largely as a result of very few studies that examined athletes, and only one study that investigated transfer to sport tasks.

## Task Representativeness

When it comes to the representativeness of perceptual training interventions, Renshaw et al. provide a commentary that highlights the necessity to couple perception, cognition and action during training, and critically reviews studies of brain training and perceptual-cognitive training. In sum, they propose a theoretical framework to address these issues by emphasizing the inter-relation between motor processes, cognitive and perceptual functions as well as the constraints of the sport task to be learned.

In a field study, Maloney et al. used a mixed-methods approach to compare the affective and cognitive demands of training and competition in elite Taekwando athletes. They found that the demands of training failed to replicate those of competition, questioning the usefulness of existing training paradigms.

Finally, van Maarseveen and Oudejans studied kinematics and gaze behavior in contested and uncontested basketball jump shots and found significant differences across the two, highlighting the need to include contested shots during jump-shot training. Moreover, *post-hoc* splits of the sample indicated that the better athletes showed more stable gaze behavior than the athletes with worse performance.

## Perception-action Coupling

The final three studies examined how the degree of coupling between perception and action influenced anticipatory performance and motor learning. Unenaka et al. investigated the effect of concurrent movement during an action-prediction task in basketball free throws. The results showed that only less-skilled athletes exhibited enhanced prediction accuracy, but skilled athletes did not, in an imitative-motion condition which required synchronous right-wrist flexion.

Fukuhara et al. investigated whether the slow-motion presentation of tennis forehand strokes would improve anticipatory judgements of shot direction and position recognition in skilled and novice tennis players. In contrast to expectations, only minor effects were revealed with the highest recognition performance for the experts in the slowest replay speed which, however, was not related to anticipation performance.

Finally, Klostermann and Hossner attempted to tackle the strong but also paradox finding of longer final fixation durations (i.e., Quiet Eye, QE) in experts. To this end, a motor learning study was conducted with the prediction that a large degree of variation in the task during learning would require longer QE durations in post- and retention tests. However, this was not the case, suggesting that rather a small but very dense amount of movement experience required descriptively longer QE durations.

Taken together, this Research Topic demonstrates the impressive breadth of research currently being undertaken but also provides a reminder of the work to be done to develop and test more representative training methods and a more common methodological design to improve our understanding of the optimal means by which to facilitate the acquisition of perceptual-cognitive skills in sports.

## Author Contributions

All authors listed have made a substantial, direct and intellectual contribution to the work, and approved it for publication.

### Conflict of Interest

The authors declare that the research was conducted in the absence of any commercial or financial relationships that could be construed as a potential conflict of interest.
